# Acute syphilitic posterior placoid chorioretinitis: when the great mimicker cannot pretend any more; new insight of an old acquaintance

**DOI:** 10.1186/s12348-022-00286-2

**Published:** 2022-02-22

**Authors:** Piergiorgio Neri, Francesco Pichi

**Affiliations:** 1 The Eye Institute Cleveland Clinic Abu Dhabi, Al Maryah Island−Abu Dhabi, PO Box 112412 UAE; 2 Cleveland Clinic Lerner College of Medicine, Case Western Reserve University, Cleveland, USA; 3 Khalifa University, Abu Dhabi, UAE

**Keywords:** Syphilis, Pre-retinal precipitates, Multifocal retinitis, Retinochoroiditis, Posterior uveitis

## Abstract

**Purpose:**

To review the multimodal imaging patterns of Acute Syphilitic Posterior Placoid Chorioretinitis (ASPPC).

**Methods:**

A systematic review.

**Results:**

Syphilis has started to attract the attention of researchers once again due to recent surges, with The World Health Organization (WHO) reporting around 12 million new cases per year. When left untreated, syphilis has a mortality rate of 8–58%, with a higher death rate in males. Eye manifestations occur both in secondary and tertiary stages of syphilis, although ocular involvement may occur at any stage of the disease.

Syphilis has been always recognized as “the great mimicker” since it can have multiple clinical patterns of presentation.

However, Acute Syphilitic Posterior Placoid Chorioretinitis (ASPPC) represents the typical pattern of the disease and can be easily distinguished.

In addition, the advent of modern technologies and the progress made in multimodal imaging have provided more details on its identikit: the pattern of pre-retinal, retinal, retinochoroidal and optic nerve involvement can be identified before going through the laboratory work-up for a correct and appropriate investigation of the disease.

**Conclusion:**

This review highlights the peculiar pattern of ASPPC, by reporting the diagnostic process made by all the imaging techniques used for a correct multimodal imaging assessment.

## Introduction

Syphilis is certainly one of the leading causes of uveitis [1, 2]. The surge reported in 1990 recorded 20.3 cases per 100,000 population [3] which is the highest incidence per annum reported in the United States of America till now. After that surge, both primary and secondary syphilis cases dropped down to 2.1/100,000 population in 2000,equal to 89.7% drop down compared to the preceding decade [3]. Although the Centers for Disease Control (CDC) aimed to eradicate syphilis, in the following years cases both of primary and secondary syphilis ramped up once again [4] doubling its prevalence in 2010 [5, 6].

*Treponema pallidum,* which is the syphilis bacterium, has a thin and elongated structure (6–15 μm), which slowly grows and may chronically infect the host. The bacterium colonizes the host through skin microlesions as a consequence of unprotected sex activity. Congenital syphilis represents the exception to that rule, since the pathogen follows the maternofetal transmission during pregnancy. The infection has a long incubation due to the slow growth, occurring within 3 weeks since the exposure. Consequently, the primary lesions are generated right at the site of inoculation [6] initiating the phases of the disease. if a prompt action is not taken, the bacterium spreads via the bloodstream towards the central nervous system (CNS) where it triggers the local immune system generating a consequent neurodegeneration.

Syphilis has a broad spectrum of clinical presentations during its early stage. Once a patient has sero-reactivity without other evidence of primary, secondary, or tertiary disease, this phase takes the name of latent syphilis [6].

In primary syphilis [6] the skin lesion at the inoculation site is the hallmark of the disease. Afterwards, it evolves into an ulcerative solitaire, clean-based and indurated wound occurring 2–3 weeks after the agent’s exposure.

Consequently, secondary syphilis [6] is generated by hematogenous spreading of the bacterium: it presents a broad range of clinical manifestations, such as sore throat, headache, myalgia, low-grade fever, and the typical copper colored macular rash affecting hands palms or soles of the feet. If either misdiagnosed or overlooked, secondary syphilis lesions may spontaneously resolve.

If no treatment is given, secondary syphilis would progress into a further stage called “latent”. At this stage, only laboratory tests may confirm a possible suspect of infection [6] due to the aspecific clinical symptoms.

However, the so called neurosyphilis may occur at any time during the infection’s course [6].

### Ocular manifestations overview

Eye involvement is more likely to happen during secondary and tertiary syphilis, even though this may occur at any time [7].

Albeit syphilis may affect any component of the eye structure, anterior segment seems to be less likely to be involved while the posterior pole offers a broad spectrum of possible clinical patterns [8].

**Fig. 1:**
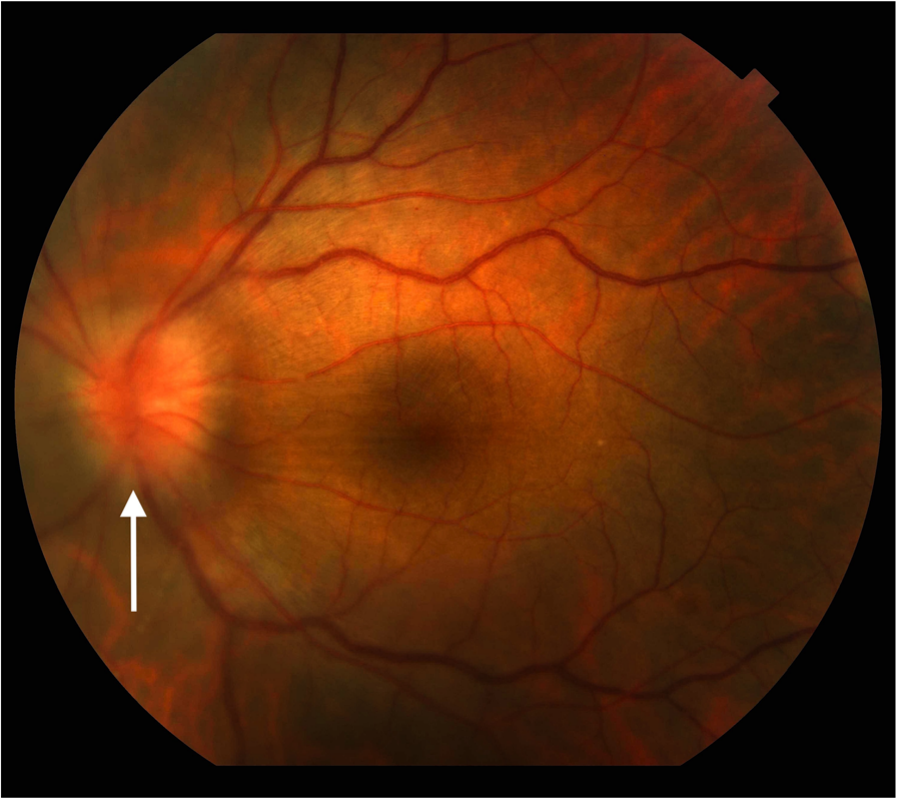
Fundus color picture of tertiary syphilis: note the optic nerve swelling (white arrow)

*Treponema pallidum* is capable to affect all the retinal layers [9, 10], and that explains why a specific pattern is often not clearly recognized. Posterior uveitis is the most typical clinical presentation, albeit syphilis may present a broad spectrum of clinical phenotypes: Syphilis has been called for many decades “the great mimicker” just for that reason. Even though syphilis may have multiple posterior pole manifestations, retinochoroiditis is the commonest [8], often presenting like a macular placoid lesion. The discrimination between acute and chronic syphilitic posterior uveitis is crucial for the decision making and the long-term outcome: in the acute phase the uveitis is florid, it progresses rapidly, often associated with meningeal involvement [11]. Chorioretinitis, neuroretinitis, retinochoroiditis, as well as retinal necrosis [12–14] represent other possible patterns of presentation. Vitreous involvement might be significant as well as papilledema (Fig. 1). Visual acuity might rapidly worsen [13], unless a rescue penicillin treatment is promptly administered. Chronic posterior syphilitic uveitis [12] will always represent a challenge: often subtle, it has a synchronous presentation with subclinical neurosyphilis. A modest vitreous involvement with mild retinal pigment epithelial inflammation represent a typical hallmark. Multifocal choroiditis is often one of the clinical presentations, while low grade retinal vasculitis is observed in the majority of the patients.

**Fig. 2:**
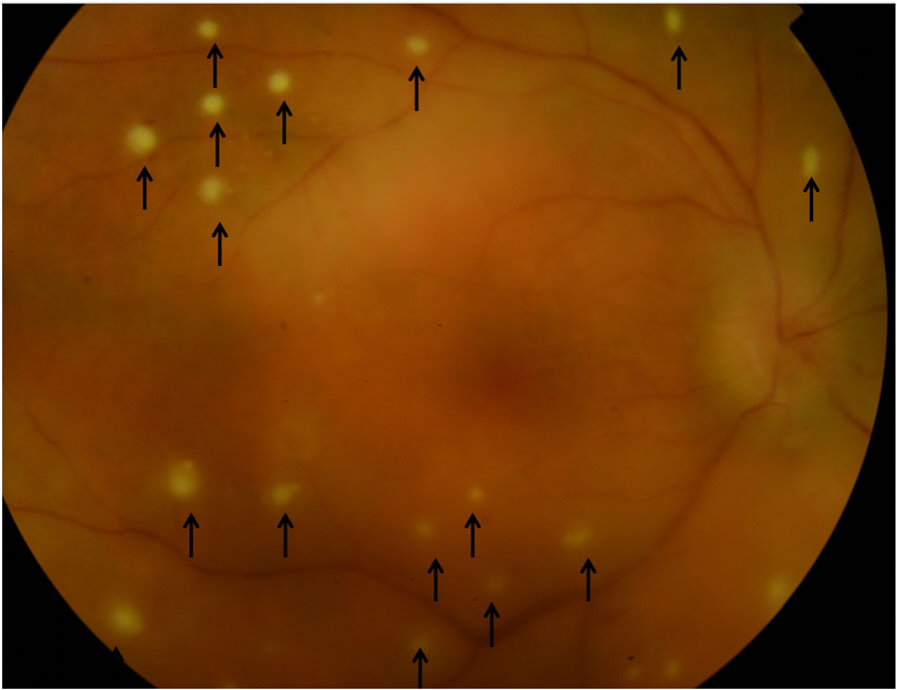
Fundus color picture showing pre-retinal lesions overlying active retinitis (black arrows) associated with vitritis

**Fig. 3:**
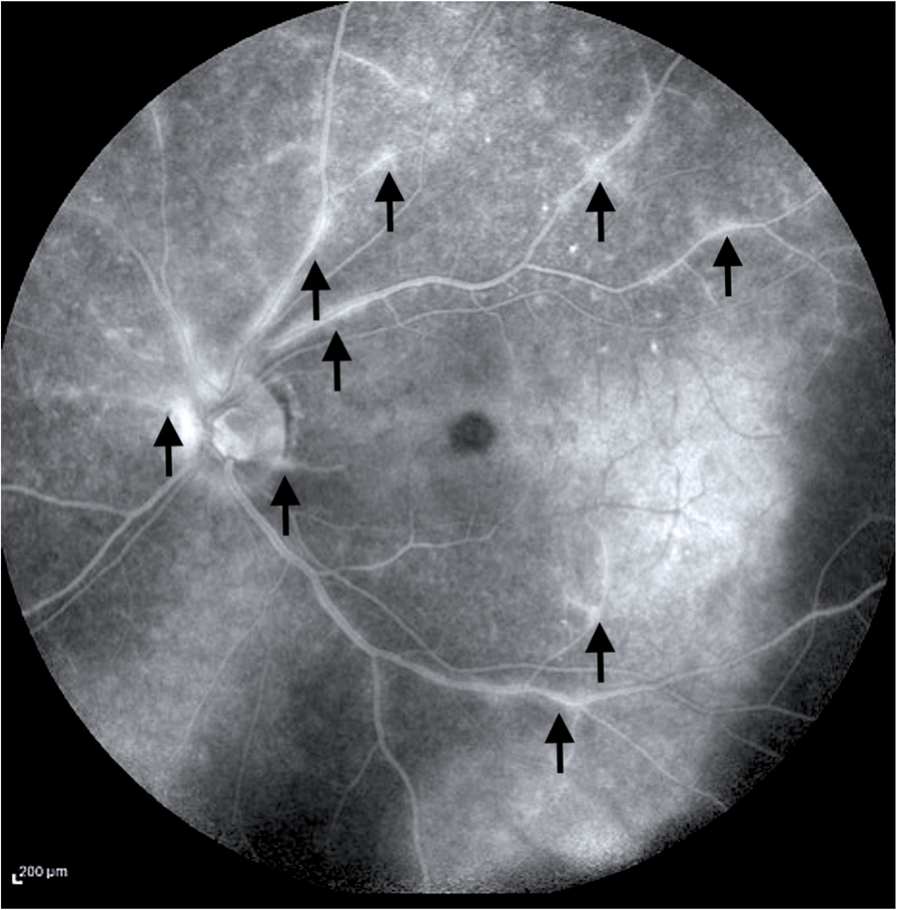
Fundus fluorescein angiography in a case of tertiary syphilis, revealing segmental leakage typical of retinal vasculitis (black arrows)

Pre-retinal lesions (Fig. 2), used to be called pre-retinal precipitates [14–16], are dots overlying an active retinitis. Authors pictured those lesions as aggregates of white blood cells [14], since often associated with a significant vitritis [15]: the hypothesis was a possible migration of such cells across the inflamed retina. Punctuate retinitis represents a hallmark of ocular syphilis: OCT assessment may show a typical pattern leading to a prompt and correct diagnosis. Fluorescein angiography (FA) contributes for sure in revealing the extension and characteristics of the associated retinal vasculitis [17, 18] (Fig. 3). An occlusive arteriolitis may lead to frosted branch angiitis, and/or presence of Kyrieleis plaques. Cystoid macular edema is often observed. Phlebitis [19] is very common in syphilis too [17–19]. Multifocal retinitis might represent an eventual expression of syphilis [20], where the intra-retinal foci are well visualized by SD-OCT which may distinguish such lesions from the superficial ones earlier reported. Poor visual outcome is an eventuality due to the deeper retinal involvement [20] and a prompt rescue treatment of penicillin must be initiated.

### Acute syphilitic posterior placoid chorioretinitis (ASPPC)

Syphilitic placoid retinochoroiditis is certainly the most typical, although the confluent pattern might rarely occur [21]. The placoid form is characterized by a ground-glass pattern [22], easily differentiated from the typical whitish necrotic lesions of both herpes and toxoplasma gondii [23].

**Fig. 4:**
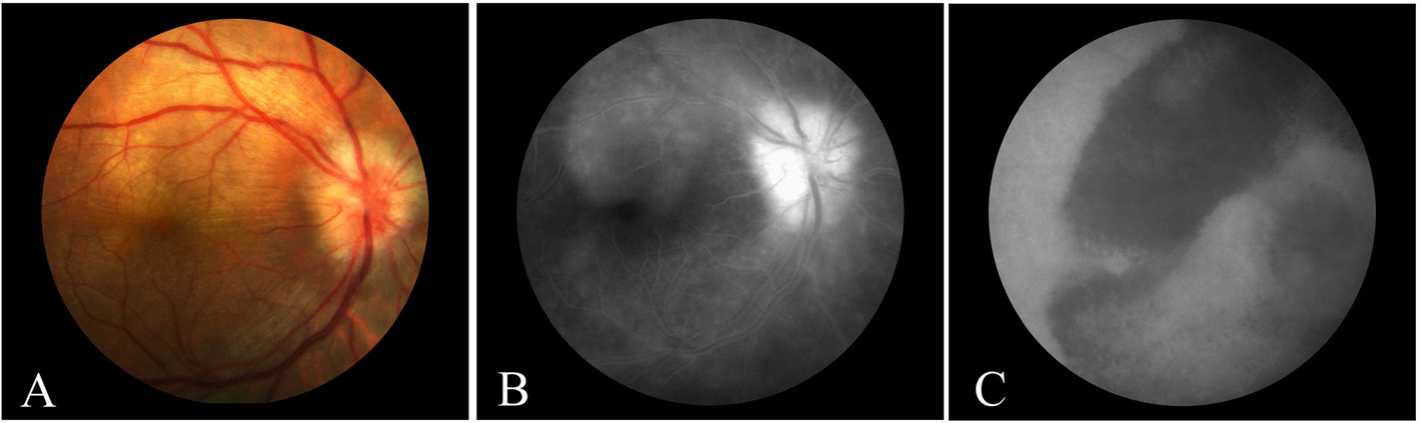
Fundus color picture showing a typical large, roundish, yellowish, placoid lesion affecting the RPE within the macular are, hallmark of ASPPC (**A**). At fundus fluorescein angiography, the macular lesion corresponds to an hyper-fluorescent area (**B**), while indocyanine green angiography presents an obvious hypo-fluorescence matching the FFA findings (**C**)

Albeit de Souza et al. [24] reported in 1988 three cases of chorioretinitis as early onset secondary syphilis, however Gass et al. [25] coined the term which is now broadly utilized to describe this clinical pattern. Acute syphilitic posterior placoid chorioretinitis (ASPPC) well describes the large, roundish, yellowish, placoid lesion occurring at level of the retinal pigment epithelium (RPE) at the macular/paramacular area (Fig. 4A). ASPPC is most peculiar clinical pattern observed in patients with concomitant HIV infection [26]. The pattern of presentation is explained by the fact that *T. pallidum* affects the choroid via blood stream and, consequently, it invades the outer retina of the macula [27]. Many cases have so far been reported in medical literature since its first description [27].

### Fluorescein angiography

Fluorescein angiography (FA) shows a progressive hyperfluorescence within the involved area, seldom presenting scattered focal hypofluorescence, or leopard spotting like appearance (Fig. 4B) [27, 28]. A further increase in leakage at the late phase [28] may reveal a neighboring active leading edge.

### Indocyanine green angiography

Indocyanine green angiography (ICGA) shows hypofluorescent areas (Fig. 4C) variable in their extension: it has been hypothesized that this may be due to choriocapillaris hypoperfusion and/or blockage of the choroidal fluorescence by the overlying affected RPE [29]. The extension of the lesions at ICGA corresponds to the areas observed at FA, even though the edges might be better determined at ICGA exam.

### Fundus autofluorescence

**Fig. 5:**
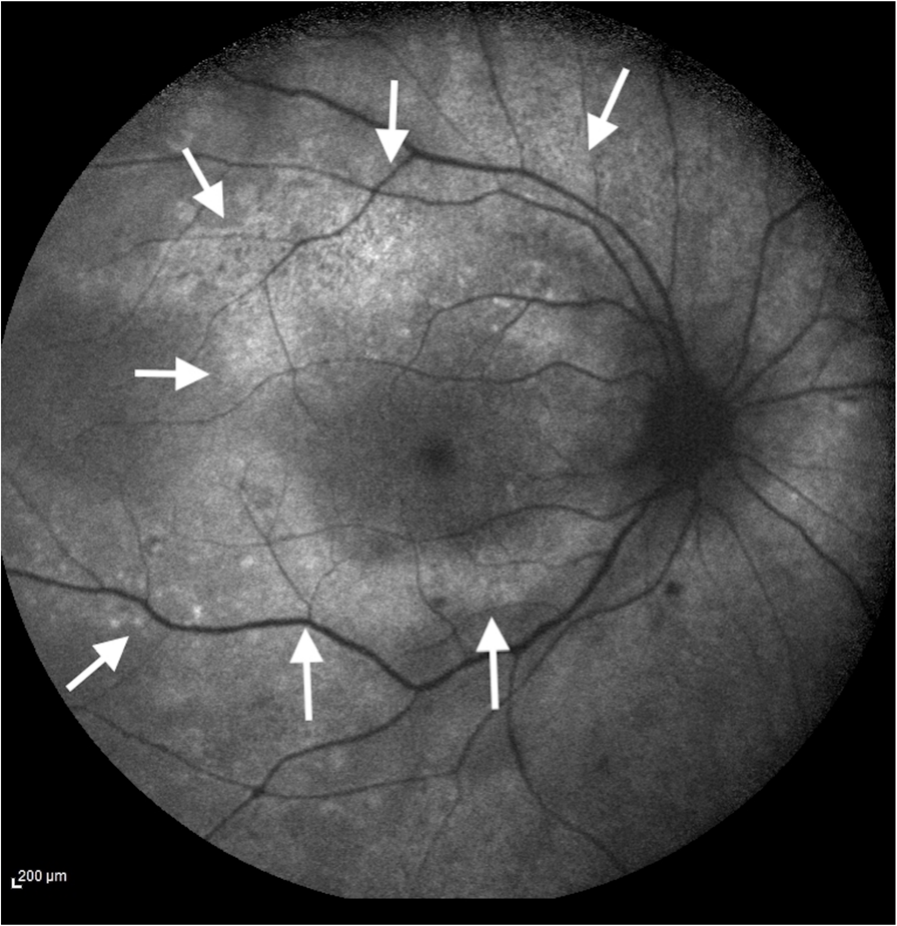
Fundus autofluorescence showing a broad hyper-autofluorescence at the macular area (white arrows)

Fundus autofluorescence (FAF) shows an obvious hyperautofluorescence (Fig. 5), often associated with tiny hyperautofluorescent dots representing RPE–photoreceptor complex material overlying the RPE due to an impaired metabolism of RPE itself [21, 26].

### Optical coherence tomography

Spectral domain optical coherence tomography (SD-OCT) is undisputedly the gold standard for ASPPC assessment and diagnosis. As we previously stressed, clinical hallmarks of ASPPC are very typical, showing an obvious choriocapillaris-RPE complex involvement.

**Fig. 6:**
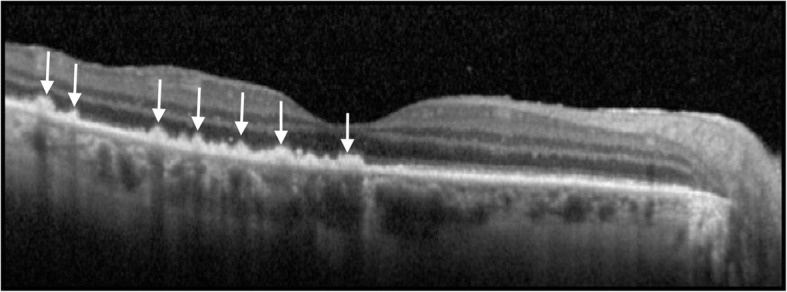
Spectral domain optical coherence tomography of a tertiary syphilis showing intact external limiting membrane, disrupted ellipsoid zone, thickened and granular hyperreflective RPE, with nodular elevations (white arrows)

**Fig. 7:**
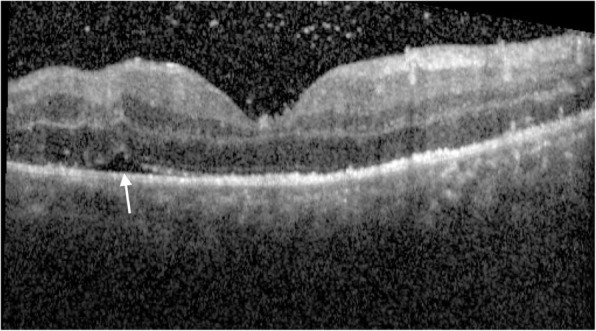
Spectral domain optical coherence tomography of a secondary syphilis showing the presence of sub retinal fluid (white arrow) associated with thickened neurosensory retina and RPE–choriocapillaris complex

Originally, Joseph et al. [29] described two cases affected by ASPPC. The authors used a time domain OCT (TD-OCT) which is certainly a low-quality technology compared to SD-OCT, not available at that time. They studied patients at the onset of their symptoms, reporting the presence of sub retinal fluid (SRF) associated with thickened both neurosensory retina and RPE–choriocapillaris complex. Lately, other authors described similar OCT findings without any evidence of SRF [27–31]. Eandi et al. [27] described the TD-OCT findings of 8 eyes with ASPPC, presenting with a shallow contour of the retina without either neurosensory retina or RPE detachment. However, the overall prevalence of SRF in hyper-acute ASPPC in 11/93 eyes (11.8%) [27]. SD-OCT findings of ASPPC with transient SRF in patients within the first 2 days of disease onset (43.3%) were reported by Pichi et al. [28]. The clinical hallmarks were an intact ELM, disrupted EZ, thickened and granular hyperreflective RPE, without nodular elevations (Fig. 6). Albeit the incidence of SRF (Fig. 7) in Pichi’s cohort was somehow higher than Eandi’s series, we hypothesized that earlier studies overlooked minimal SRF in the acute phase, according to the lower resolution of TD-OCT.

Sub-foveal RPE-choriocapillaris complex appear thickened and hyperreflective in the acute phase of ASPPC. Irregular nodular, hyperreflective elevations at the RPE and photoreceptors’ junction, associated with segmental loss of the ellipsoid band seems to be the natural evolution within 1 week after presentation, albeit with no evidence of new or persistent sub-foveal fluid being observed [2].

Brito et al. [30] reported an acute loss of choroidal vascular structure. In the series by Pichi et al. [28], hyperreflective spots in the choroid were described in 30.8% of the eyes, at days 1 to 2 and were persistent at days 7 to 9. In addition, scattered hyperreflective spots in the choroid were described in 10 eyes also.

Systemic penicillin treatment led to the normalization of the outer retinal and choroidal structure, as well as an obvious improvement of the visual acuity. One-month antibiotic therapy lead to a complete restoration of EZ band with normalization of the RPE at SD-OCT, as well as lack of hyperreflective spots at the choroidal level.

Since patients with ASPPC usually receive prompt antibiotic treatment after serologic investigation, very few reports on the natural course are available. However, in 2014 Armstrong et al. [31] reported the natural course of ASPPC to chorioretinitis: ASPPC evolved into panuveitis within 6 weeks after the initial diagnosis. No spontaneous resolution of the lesion was observed. This peculiarity might suggest ASPPC as an early stage of posterior uveitis. Franco et al. [32] described a spontaneous recovery of the outer retina changes on SD-OCT within 2 weeks. Baek et al. [33] reported a similar untreated case of ASPPC with spontaneous resolution and solved macular placoid anomaly bilaterally. SD-OCT showed granular hyperreflective spots at RPE level. However, the longer follow-up compared to Franco et al. [32] showed a progressive worsening of posterior uveitis. Aranda et al. described a case of ASPPC [34] with spontaneous healing. Such an event might be interpreted as a potential response of the regional immune system to the pathogen. Syphilis characteristically presents episodes of active disease alternated with periods of latency. A further hypothesis is represented by the spontaneous regression of ASPPC determined by the prolonged latency induced by the host’s reginal immune response.

We speculated that choroidal hyperreflective spots seen on SD-OCT might represent inflammatory foci in the choroid vasculature since the circulating *T. pallidum* might enter the outer retina space via choroid.

Antibodies might react with treponemes in the choroid and lead to the RPE-choroidal involvement. High levels of anti-beta2 glycoprotein antibody were reported by Brito et al. [30] in a case of ASPPC. Such antibodies might lead to regional choroidal occlusive events and, consequently, to altered RPE metabolism with disorganization of RPE structure itself: that might be the possible pathophysiology of hyperreflective nodularity at SD-OCT. Hyper-acute disruption of the outer blood ocular barrier might also produce variable amounts of SRF.

The reports of spontaneous resolution of ASPPC with late-onset posterior uveitis [32] opens a discussion on a possible role of the immune system as a modulator of the immune-phenotype of syphilis.

### Laboratory evaluation

The laboratory assessment of syphilis counts 2 main typologies of tests [35]: one has the detection of patients with possible infection as the primary goal, while the second aims to validate the test results and minimizes possible false positives.

The first method for syphilis infection analysis is represented by non-treponemal tests: antigens extracted from normal mammalian tissues react with antibodies versus *T pallidum* bacteria [36]. Rapid plasma reagin (RPR) and venereal disease research laboratory (VDRL) tests aim to quantify both IgG and IgM antibodies [37]. The titers resulting from non-treponemal antigen tests present a precise correlation with syphilis activity. The titers of non-treponemal antibody tend to reduce according to treatment efficacy. Anti-syphilis therapy is considered significantly effective when the titer of the antibody is reduced by a fourfold, while lack of reduction or an increase of the same titer is interpreted either as a treatment failure or a possible re-infection [38].

It is crucial to remark that 30% of patients in latent or tertiary stages may present negative non treponemal tests [36].

Consequently, it is intuitive that specific treponema antibody assay should be integrated to non-treponemal tests, in order to investigate all the cases of suspected disease [37]. Fluorescent treponemal antibody adsorbed (FTA-ABS) tests and *Treponema pallidum* particle agglutination (TPPA) are specific treponemal tests, aiming to detect antibodies to treponemal antigens. Less expensive, user friendly, and automatable treponemal tests such as enzyme immunoassays are now commonly used in most laboratories. Treponemal tests offer a qualitative instead of a quantitative analysis, albeit often they remain positive life long, despite an effective treatment and consequently, they do not offer advantages in testing the response to treatment. However, since the number of cases increased tremendously in the recent past leading to a considerable escalation of the costs, reverse algorithm testing was proposed in order to implement sustainability.

As a consequence of that, treponemal tests are the first choice, typically IgG detection by EIA [38], followed by the evidence that false positive treponemal tests present a lower rate compared to the false positive non-treponemal tests. Cases presenting a positive result to a treponemal screening test should undergo a non-treponemal test. When a non-treponemal test turns negative, the laboratory should validate the result by using a different treponemal test in order to check and confirm the first result. In case of a second positive treponemal test, patients previously treated will not need further therapies, unless they will undergo further exposure or will present potentially harmful habits. Patients with no history of any anti-treponemal treatment should receive treatment as appropriate, while in case of second negative treponemal test no further evaluation or treatment should be considered [39]. Reverse algorithm testing offers an excellent cost/benefit profile for screening low-prevalence populations, albeit the head-to-head comparison reverse algorithm showed 6/1000 false-positive tests while traditional algorithm had not one [39, 40].

However, CDC still recommends traditional RPR-based screening algorithm instead of the new approach.

In case of dubious results, CDC suggested that selected patients should receive lumbar puncture for the diagnosis of syphilis [40] on the basis of the following criteria:

1. Central nervous system, ocular or auditory involvement raising the suspect of active tertiary syphilis
2. Evidence of treatment failure with long-lasting quadruple VDRL or RPR increase, or elevated RPR titer (> 1:32) that does not decrease 2 titers for 6–12 months in early syphilis or 12–24 months in latent syphilis.

Furthermore, CDC mandate lumbar puncture in cases affected by ocular syphilis in order to rule out a possible CNS involvement, albeit questionable if only isolated anterior segment inflammation happens [40].

VDRL represents the standard, highly specific serological test for CSF analysis [41], while non-treponemal tests do not represent an option.

On the other hand, European Guide Lines suggest a different approach [42]. A specific treponemal tests screening algorithm is preferred versus non treponemal tests, particularly by well-equipped European laboratories. This algorithm has a design for a favorable cost/effectiveness, particularly for automated screening in high volume facilities, such as blood/plasma donors in asymptomatic populations. The algorithm distinguishes patients successfully treated for syphilis and those who were not. Treponemal tests give also a higher sensitivity in detecting very early syphilis screening compared to non-treponemal tests. On the other hand, a higher number of false-positive tests might occur in populations with a lower prevalence.

However, a screening algorithm privileging non treponemal tests is still recommended in some countries. This specific recommendation is indicated in order to detect the prozone phenomenon in infectious syphilis: consequently, a quantitative test is preferred. This specific algorithm aims to detect only active syphilis, although it presents a lower sensitivity compared to the algorithm privileging the treponemal tests: this results in a lower ability in detecting very early syphilis.

The algorithm combining treponemal tests and non-treponemal tests is particularly indicated when there is high suspicion of very early syphilis. This method may be recommended when history of contacts of syphilis cases or peculiar signs such as recent gangrene are reported: this may reveal those cases reacting to non-treponemal tests which may become as such before treponemal tests.

### Treatment

Ocular syphilis treatment protocol recommended by CDC does not differ from the one used for neurosyphilis [43]. Aqueous penicillin G or procaine penicillin G plus probenecid are the first line treatment for ocular syphilis [44], due to the poor penetration of benzathine penicillin into the blood ocular barrier. In case of CNS involvement, the treatment regimen is the following: fractionated 18–24 MU /day of intravenous aqueous penicillin G administered every 4 h for 10–14 days. In addition, a group of key opinion leaders suggest to add 2–3 doses of benzathine benzylpenicillin intravenous treatment [44].

If allergic reaction to penicillin is reported, treatment strategy presents challenges: aminoglycosides, fluoroquinolones, and sulphonamides antibiotics have no efficacy, while doxycycline might represent a possible alternative for early and late latent syphilis [45]. Azithromycin also has shown a promising efficacy in treating early syphilis (single 2-g oral dose) [46]. However, *T. pallidum* chromosomal mutations associated with macrolide, including azithromycin, lead to both resistance and treatment inefficacy as per the reports in several sites in the United States.

The European guidelines [42] offer a precise and tailored approach by differentiating the stages and providing a pragmatic method which considers also the fact that patients may refuse parenteral treatment at some point. In case of primary, secondary and early latent syphilis, intramuscular penicillin benzathine G is indicated as first line treatment at the dose of 2.4 MU, administered either a single injection or as two injections of 1.2 MU. Due to the pain provoked by penicillin benzathine G injection, 0.5–1 cc of the diluent might be replaced by lidocaine 1% solution without epinephrine. A period of 30 min observation is strongly recommended for clinical review after injection. Unfortunately, penicillin benzathine G may have shortages and supply disruptions, despite several companies produce it in Europe and worldwide. Procaine penicillin 600,000 units intramuscularly per day for 10–14 days is a second-line therapy option. In case of bleeding disorders, 1 g of intravenous Ceftriaxone in a single daily dose for 10 days might be an option. If either penicillin allergy is reported or intravenous treatment is refused, oral doxycycline at the dose of 200 mg daily for 14 days represents a valid alternative.

In case of Late latent, cardiovascular and gummatous syphilis the first-line therapy option is still penicillin benzathine G at the dose of 2.4 MU, given at day 1, 8 and 15. Intramuscular procaine penicillin at a dose of 600,000 units per day for 17–21 days might be considered, if penicillin benzathine G is either not available or specific medical reasons are reported. Once again, oral doxycycline 200 mg daily for 21–28 days represent a valid alternative for the same reasons we reported here above.

A specific part of treatment has to be dedicated to steroids therapy which plays a crucial role on the economy of the therapeutic outcome.

Steroids represent an essential adjuvant therapy for patient presenting ocular syphilis. When syphilitic anterior uveitis, keratitis and scleritis are present, topical steroids are a necessary adjunctive therapy. Systemic steroids do not represent only an important component for the treatment of posterior uveitis and optic nerve inflammation [42], but also for the prevention of Jarisch-Herxheimer reaction (JHR), occurring in 30–50% of treated cases [47].

As Solebo AL and Westcott M [48]. remarked, once an appropriate treatment is started, a definite role exists for adjunctive oral or intravenous corticosteroids in syphilitic optic neuritis, posterior uveitis, as well as scleritis.

Although several reports addressed the importance of prednisolone as an adjuvant treatment, no consensus might be found in the medical literature in its use for ocular syphilis. Prednisolone has been used in the past to prevent febrile episodes [49].

Although empirical and not scientifically proven, steroids appear biologically plausible to help in preventing possible issues to optic nerve and uveal tract due to JHR. Prevention of JHR itself represents the only condition addressed by the European guidelines: 20–60 mg per day of oral prednisolone for 3 days, given 24 h before commencing specific syphilis treatment are recommended [42] to lower the risk of JHR occurrence.

## Conclusions

Ocular syphilis represents a challenge for both retina specialists and uveitis specialists: the variety of clinical phenotypes is always hard to distinguish. However, the modern concept of multimodal imaging methodology has given sufficient tools to physicians for the correct interpretation of clinical findings. ASPPC represents a typical expression of syphilis which can be correctly addressed by accurately analyzing the OCT findings which appears almost exclusively related to ocular syphilis. On the other hand, further appropriate and specific tests, such as treponemal and non-treponemal tests, are necessary to confirm the disease and consequently start an efficient treatment strategy.

### Compliance with ethical standards

This study received no funding.

This article does not contain any studies with human participants or animals performed by any of the authors.

## Data Availability

N/A
